# A Novel Ship Detection Method Based on Gradient and Integral Feature for Single-Polarization Synthetic Aperture Radar Imagery

**DOI:** 10.3390/s18020563

**Published:** 2018-02-12

**Authors:** Hao Shi, Qingjun Zhang, Mingming Bian, Hangyu Wang, Zhiru Wang, Liang Chen, Jian Yang

**Affiliations:** 1 Department of Electronics, Tsinghua University, Beijing 100084, China; shihao@tsinghua.edu.cn (H.S.); yangjian_ee@tsinghua.edu.cn (J.Y.); 2 Beijing Institute of Spacecraft System Engineering, Beijing 100094, China; bianmingming2008@163.com; 3 Department of Information and Electronic, Beijing Institute of Technology, Beijing 100081, China; whyholly@163.com (H.W.); 2220170436@bit.edu.cn (Z.W.)

**Keywords:** ship detection, Haar-like feature, integral image, single-polarization SAR image, Gaofen-3

## Abstract

With the rapid development of remote sensing technologies, SAR satellites like China’s Gaofen-3 satellite have more imaging modes and higher resolution. With the availability of high-resolution SAR images, automatic ship target detection has become an important topic in maritime research. In this paper, a novel ship detection method based on gradient and integral features is proposed. This method is mainly composed of three steps. First, in the preprocessing step, a filter is employed to smooth the clutters and the smoothing effect can be adaptive adjusted according to the statistics information of the sub-window. Thus, it can retain details while achieving noise suppression. Second, in the candidate area extraction, a sea-land segmentation method based on gradient enhancement is presented. The integral image method is employed to accelerate computation. Finally, in the ship target identification step, a feature extraction strategy based on Haar-like gradient information and a Radon transform is proposed. This strategy decreases the number of templates found in traditional Haar-like methods. Experiments were performed using Gaofen-3 single-polarization SAR images, and the results showed that the proposed method has high detection accuracy and rapid computational efficiency. In addition, this method has the potential for on-board processing.

## 1. Introduction

Automatic ship target detection technologies based on remote sensing images play a significant role in many applications, such as ocean monitoring, shipping traffic management and maintenance of maritime rights and interests. Synthetic Aperture Radar (SAR), as an active microwave imaging sensor, has the characteristic of high-resolution imaging in all-weather and all-day scenarios compared to passive sensors like optical sensors [1]. Thus, SAR images provide information services and provide decision-making support for ocean information applications.

Ships are used in many areas of human activity, and artificial interpretation is difficult in remote sensing images of large fields. For this reason, there is a need for a method of automatic ship detection. However, due to disturbances in artificial landforms, reefs and huge waves, automatic ship detection in SAR images is a big challenge [2].

Consequently, many researchers have been constantly trying to find new methods. The research shows that ship targets appear as a cluster of high-brightness pixels in SAR images. The artificial targets contain a large number of dihedral angles and have a high backscattering coefficient compared to the sea background. By utilizing this difference in amplitude distribution, a category of Constant False-Alarm Rate (CFAR)-based methods is proposed and widely used. In these CFAR methods, the center pixel is compared with a threshold within a sliding window to determine whether the pixel belongs to the ship target. The threshold is determined by statistical characteristics in the boundary ring of the focusing window under a given false alarm rate [3,4,5]. 

One of the CFAR methods is called the parametric CFAR detection method. In the parametric CFAR method, the detection threshold is determined by estimating the statistical model of the sea background. As early as the 1990s, Novak et al. [6] put forward a two-parameter CFAR, assuming that the sea background clutter in the SAR image obeys a Gaussian distribution. However, this assumption is only valid for low-resolution SAR images and homogeneous clutter. With the increase in resolution, the researchers proposed a series of statistical models to fit a heterogeneous clutter description, such as a log-normal distribution [7], Gamma distribution [8], Weibull distribution [9] and K-distribution [10]. Among them, the K-distribution provides better performance in the ocean monitoring workstation (OMW) system [11,12]. As the resolution of SAR becomes higher and higher, the K-distribution model is not always a good fit [13]. Therefore, Qin and Gao et al. [14,15] proposed a CFAR target detection algorithm based on a generalized gamma distribution. It can be adapted to many scenes of high-resolution SAR images and shows better performance than many classical parametric distributions in most cases.

Another type is the nonparametric CFAR detection method. The probability density function of the sea background amplitude is not properly fitted by a single parameterized mode when the scene of the SAR imagery is relatively complex. In this situation, nonparametric CFAR methods do not need to fit the background or target statistical models and estimate parameters but rather directly infer the model from the SAR imagery [16] through a non-parametric method. Gao [17] proposed a nonparametric CFAR algorithm based on Parzen kernel density estimation that is used to extract the ship target pixels from the candidate area. In the Parzen window kernel method, different kernel weighting methods are used for statistical distribution estimation. The result more accurately fits the different sea backgrounds. Lang [18] proposed a novel nonparametric sea background distribution estimation method based on an n-order Bézier curve. The proposed method is as good as a traditional nonparametric Parzen window kernel method. In addition, the time consumption is significantly improved. In the non-parametric CFAR method, the bandwidth of kernel density estimation (KDE) is determined empirically, which is proven to be inappropriate. Tian [19] proposes an adaptive KDE bandwidth estimation method. This method provides an automatic training sample selection scheme, which avoids the manual intervention in conventional methods.

In addition, new methods based on CFAR have been proposed. Wang [20] proposed an intensity-space domain CFAR method for ship detection. The original SAR image is transformed first in order to fuse spatial and intensity information into one index. By doing this, the target pixels are strengthened and easier to detect. Then, two-parameter CFAR is used for target detection on the transformed image. Dai [21] modified the standard CFAR algorithm to solve the problem of various ship sizes. This method proposed the variable guard windows generated by the target proposal generator to replace the original fixed guard windows. As a result, the performance of target detection under a multi-scale situation can be improved.

However, CFAR-based approaches also have limitations. First, the accuracy of the algorithm depends on the estimation accuracy of the background`s probability density function, and the performance is not satisfied under low-contrast conditions. Second, due to the calculation of the distribution parameter estimation, the computational burden and time consumption are increased with complex models.

Beyond the CFAR-based methods, some computer vision and machine learning methods have been introduced into ship target detection in recent years. For example, Zhai [22] and Wang [23] used the saliency model instead of the CFAR in the target region of interest (ROI) extraction step. Wang [24,25] enhanced the target and suppressed the background noise by calculating the multiscale Variance Weighted Image Entropy (VWIE) for each pixel. Further, some researchers [26,27,28] try to employ deep neural networks into ship target detection of SAR imagery. These methods have shown good detection capability, but more training data are required.

**Figure 1:**
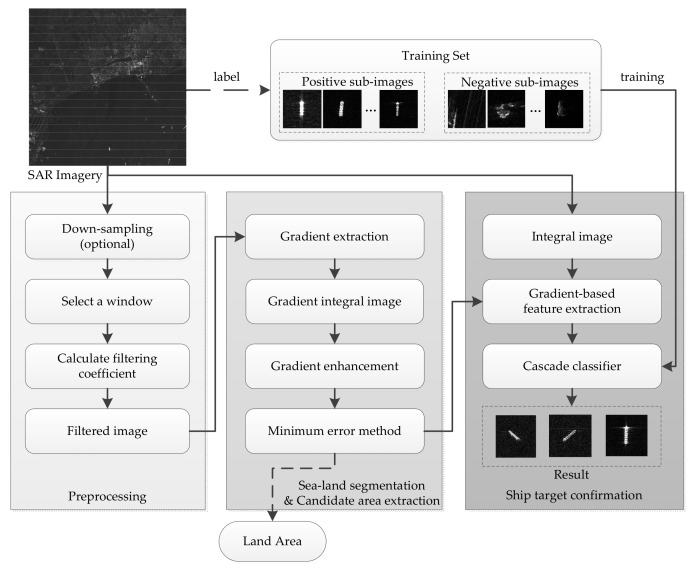
Workflow of the proposed ship detection algorithm.

This paper takes into account the radiation feature of ships in SAR images and proposes a novel automatic approach to achieve gradient and integral feature for ship detection. In this method, we not only improve the accuracy of target detection, but also concern the computation efficiency and the realization in embedded system. So we employ the linear models instead of the complex models and design the optimize computation which can be parallel reused. In order to guarantee the accuracy, we employ the classifier to identify the target. We train the classifier off-line so that improves the on-line detection effect. Figure 1 shows the algorithm workflow. This framework contains three major parts. The first one is the preprocessing step, which is focused on speckle reduction of SAR imagery. In this step, an adaptive speckle filtering method is presented. The second one is sea-land segmentation and the candidate area extraction step. In this step, we use the gradient information to segment the SAR image into land area and candidate areas. At the same time, we employ an integral graph to accelerate the calculation. The final one is the ship target confirmation step. In this step, the modified Haar-like features are proposed for describing ship characters, and finally, the target patches are identified by the Adaboost classifier. By contrast with previous work, the proposed method uses linear operations instead of transcendental functions and has the characteristic of low computation complexity and operands. In addition, the proposed method is well-adapted for multiple resolutions and situations of the SAR imagery, and it is easily implanted in an embedded system. In particular, there are many limitations in the space environment, such as on-satellite resources and power consumption. Thus, the proposed method is suitable for on-board processing.

## 2. Preprocessing of SAR Imagery 

**Figure 2:**
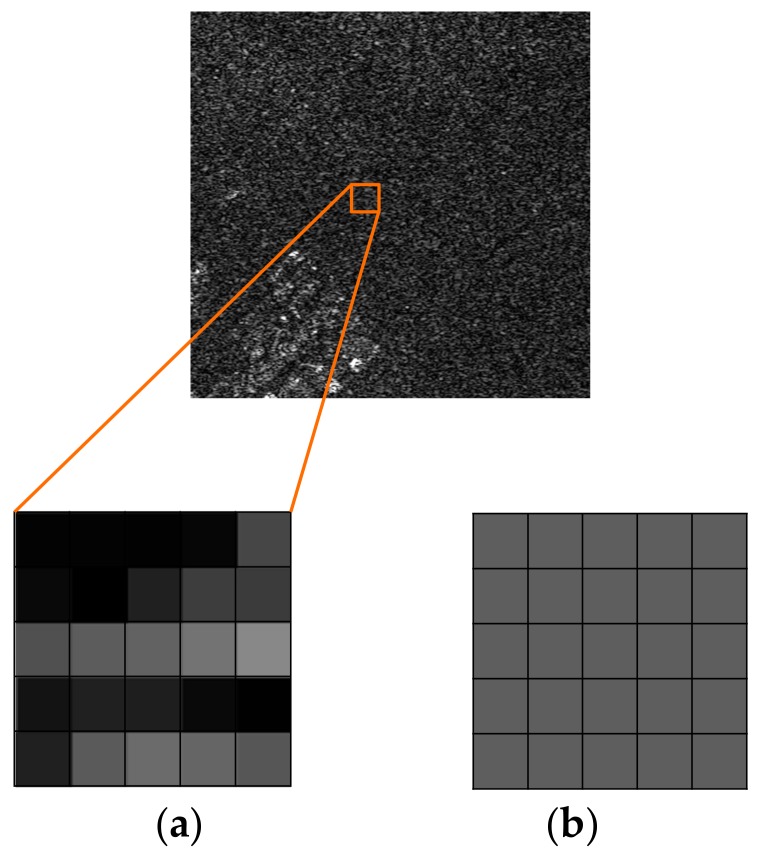
Sketch map of speckle noise in SAR imagery. (**a**) Homogeneous area in SAR imagery; (**b**) Homogeneous area in optical imagery.

Due to the different imaging theory, SAR imagery is more difficult to interpret than optical imagery. SAR, as a coherent imaging system, is based on the coherent addition of pulse echoes. In the imaging process, some kind of texture noise is inevitably generated [29]. The homogeneous areas with the same backscattering coefficient do not have the same gray level in the SAR image. The adjacent pixels have random gray values like granules, as shown in Figure 2. Therefore, the existence of speckle noise in SAR images causes many problems. For example, the intensity value of a single pixel cannot measure the reflectivity of a distributed target. Thus, the SAR imagery is unable to reflect the scattering characteristics of a target correctly, seriously affecting interpretation.

In this section, we employ a rapid adaptive filtering method to eliminate the clutters [30], while maintain the target texture information adaptively. Therefore, the filtering algorithm not only suppresses the speckle noise, but also eliminates the background clutters.

In this method, the linear models are established to accelerate the computation instead of the complex model of multiplicative noise in SAR systems. Besides, the filter parameters can be adjusted according to the image scene adaptively. Therefore, we assume the pixels around a small area have a uniform feature and employ a sliding sub-window to count the local statistic information separately.

First, we assume the *ω_k_* indicates a square window centered at pixel *k* with the width *l*. The parameter *l* influences the range of statistic pixels and the selection of parameter *l* is related to the computation cost. 

Then, to ensure the output image has the same gradient characteristic as the input image we build two linear models to describe the filter processing. Within the window, we define a linear transformation, which involves the original image ***I*** and the output image ***G***. The linear model can be shown as below:

(1) $$G_{i}=a_{k}I_{i}+b_{k},\forall i\in \omega_{k}$$

where *a_k_* and *b_k_* are linear coefficients in *ω_k_*. The *I_i_* and *G_i_* indicate the intensity of pixel *i* in the input and output image. This model ensures that the output image has the same change of gradient as the input image.

In addition, in order to achieve the effect of removing noise, we also assume that the output image can be described as input image ***I*** removing unwanted components ***e***, which indicates noise or textures. The model is shown as below:

(2) $$G_{i}=I_{i}-e_{i},\forall i\in \omega_{k}$$

In order to solve the coefficients, we need to minimize the *e_i_* in Equation (2) under the premise of Equation (1) simultaneously. So the solution is to minimize the following cost function in window *ω_k_*:

(3) $$J(a_{k},b_{k})=\underset{a_{k},b_{k}}{\min}\left\{\sum_{i\in w_{k}}\left({\left(a_{k}I_{i}+b_{k}-I_{i}\right)}^{2}+\varepsilon a_{k}^{2}\right)\right\}$$

where *ε* is a regularization parameter. We can obtain the solution as described below. 

When ***a*** is fixed, calculate the derivative of *J* with respect to *b* and make it equal to 0. Then, we can obtain:

(4) $$\sum_{i\in w_{k}}2(a_{k}I_{i}+b_{k}-I_{i})=2(a_{k}\sum_{i\in w_{k}}I_{i}+n_{k}b_{k}-\sum_{i\in w_{k}}I_{i})=0$$

(5) $$b_{k}=\frac{(1-a_{k})\sum_{i\in w_{k}}I_{i}}{n_{k}}=(1-a_{k})\mu_{k}$$

where *n_k_* is total number of pixels and *μ_k_* is the mean of *I* within *ω_k_*.

Substitute the above result into Equation (3), and then calculate the derivative of *J* with respect to *a*. Make the derivative equal to 0, we can then obtain:

(6) $$\sum_{i\in w_{k}}2\left[I_{i}(a_{k}I_{i}+b_{k}-I_{i})+\varepsilon a_{k}\right]=2(a_{k}\sum_{i\in w_{k}}I_{i}{}^{2}-\sum_{i\in w_{k}}I_{i}{}^{2}+\sum_{i\in w_{k}}I_{i}\mu_{k}-a_{k}\sum_{i\in w_{k}}I_{i}\mu_{k}+n_{k}\varepsilon a_{k})=0$$

(7) $$a_{k}=\frac{\sum_{i\in w_{k}}I_{i}{}^{2}-\sum_{i\in w_{k}}I_{i}\mu_{k}}{\sum_{i\in w_{k}}I_{i}{}^{2}-\sum_{i\in w_{k}}I_{i}\mu_{k}+n_{k}\varepsilon }=\frac{\frac{1}{n_{k}}\sum_{i\in w_{k}}I_{i}{}^{2}-\mu_{k}{}^{2}}{\frac{1}{n_{k}}\sum_{i\in w_{k}}I_{i}{}^{2}-\mu_{k}{}^{2}+\varepsilon }=\frac{\sigma_{k}}{\sigma_{k}+\varepsilon }$$

where *σ_k_* is variance within *ω_k_*. Thus, we can obtain the linear coefficients. The filtering output is the addition of different results in *ω_k_* after the sub-window sliding all over the input image, as described below:

(8) $$G=\sum_{k}\left[a_{k}I_{i}+(1-a_{k})\mu_{k}\right],\forall i\in \omega_{k}$$

where *k* is the number of the sub-window.

According to the introduction of principle deriving, the adaptive filtering method can be implemented by following steps. First, normalize the input image *I*, in order to limit each pixel value from 0 to 1.

(9) $$I_{n}=\frac{I}{\max(I)}$$

Second, take square window *W*(*x*,*y*) centered by *I_n_*(*x_c_*,*y_c_*) with width *l* overlapping the sliding image one pixel by one pixel. At the same time, calculate the mean value, mean of square value and the variance within the window as shown in the following formula:

(10) $$Mean(W)=\frac{1}{l\times l}\sum_{y=1}^{l}\sum_{x=1}^{l}W(x,y)$$

(11) $$Mean(W^{2})=\frac{1}{l\times l}\sum_{y=1}^{l}\sum_{x=1}^{l}W{\left(x,y\right)}^{2}$$

(12) $$Var(W)=Mean(W^{2})-{\left[Mean(W)\right]}^{2}$$

Third, according to Equation (7), the *a_k_* corresponding to the current pixel can be calculated. After window *W* slides the whole image, the matrix *a* with the same size as the original image can be obtained. Finally, by using Equation (8) to reconstruct the image, we can get the filtered result.

**Figure 3:**
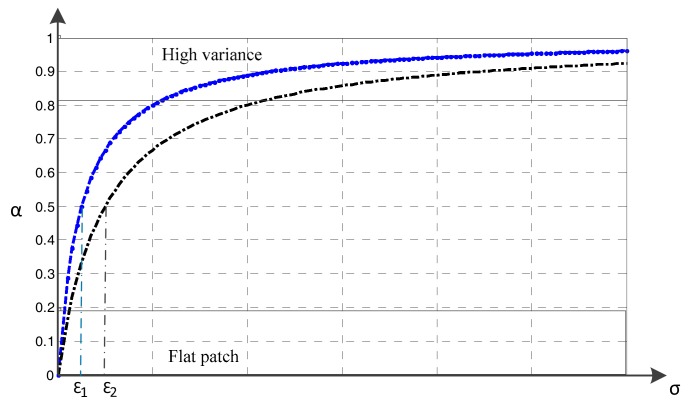
Changing trend curve of parameter *a_k_* with different *ε*. The blue curve corresponds to *ε*_1_ and the black curve corresponds to *ε*_2_.

As can be seen from the reconstruction formula, parameter *a_k_* is an adaptive weight factor to adjust the percentage between the original image and smoothed image. The adaptability of *a_k_* will be discussed later in the paper. Usually, when *ε* > 0 and is fixed, according to Equation (7), we can see that the value of *a_k_* is related to *ε* and *σ_k_*, and the relationship among them is shown in Figure 3.

When the variance is large such that *σ_k_ >> ε*, the pixel values of the image in the window undergo an obvious change. At this time, the parameter *a_k_* is close to 1, and the filtering output is almost identical to the input image, as shown in high variance area. This explains that this method will preserve the information of the original image when there is a large gradient change in the image, such as the edge information.

When the pixel values of the image in the window are relatively flat, the variance is small such that *σ_k_* << *ε*. Thus *a_k_* ≈ 0 and *b_k_* ≈ *μ_k_*, and the filtering output is the average of the pixel values in the window, as shown in flat patch area. This explains that this method will smooth the original image when there is a small gradient change in the image, such as speckle noise. From this, we can see that the variance of the sub-window determines the value of *a_k_* and the filtering effect.

In addition, the parameter *ε* influences the decision thresholds of high variance area and flat patch area. Particularly, when *ε =* 0, the parameter *a =* 1, and the filtering output is the same as the input image. When *σ_k_* = *ε*, the parameter *a* = 0.5. Thus, it can be seen from Figure 3 that when *ε* is increasing, the steepness of the curve rises slowly and the threshold of high variance significantly increases. Therefore, we can select the appropriate *ε* value according to the variation of the sub-window and expected smoothing effect. We set the parameter *ε* as 0.001–0.1 empirically.

## 3. Ship Target Detection and Identification 

Based on the filtered imagery, a ship detection strategy is proposed based on the gradient features. First, a sea-land segmentation and candidate area extraction is presented. It is used to segment and remove the disturbance of land areas and also to select the regions of interest in the target. Second, according to the edge and line features of the ship, modified Haar-like features and an Adaboost classifier are combined to identify the final target regions. Among these steps, the integral graph is employed to accelerate and simplify the calculation.

### 3.1. Sea-Land Segmentation and Candidate Areas Extraction

The filter processing reduces the speckle noise effectively and improves the visibility of the image. However, there are many complex texture structures in the land regions. Thus, masking the land regions has many benefits for detecting the ship target on the sea areas.

First, the DEM information database is widely used for distinguishing the ocean and land for large scenes. However, the limited accuracy of the DEM database is not suitable for offshore scenarios. Additionally, the junction regions of land and sea, such as the ports, are the place where ship targets appear most often. Therefore, an image-based sea-land segmentation method needs to be devised to distinguish the offshore land areas.

#### 3.1.1. Gradient Extraction

Because of the abundance of artificial targets and strong scattering points within land regions, the pixel values of SAR images will be more intense than that of sea backgrounds. Therefore, we distinguish the difference by using gradient features. Thus, we extract the gradient information through the Sobel operator.

The Sobel operator is a typical edge detection operator based on the first-order derivative, which uses a discrete differential operator to calculate the approximate gradient value. The Sobel operator consists of two 3 × 3 matrices that are horizontal and vertical templates. The convolution of these templates with image gives the corresponding gradient values. The Sobel edge detection operator template is shown below.

(13) $$S_{x}=\left[\begin{array}{ccc} -1 & 0 & 1\\ -2 & 0 & 2\\ -1 & 0 & 1 \end{array}\right],S_{y}=\left[\begin{array}{ccc} 1 & 2 & 1\\ 0 & 0 & 0\\ -1 & -2 & -1 \end{array}\right]$$

where *S_y_* represents an approximate vertical gradient template and *S_x_* represents an approximate horizontal gradient template.

Slide these templates in the image pixel by pixel, and get the convolution with the image sub-window *I_i_* to obtain the horizontal and vertical gradient. Then, select the maximum value of two directions as the gradient value of the center pixel in the sub-window, as shown below:

(14) $$I_{Sobel}=\sum_{i}\max\left(I_{i}*S_{x},I_{i}*S_{y}\right)$$

Unlike edge extraction in the optical image, the Sobel operator in SAR imagery converts the scattering properties of different objects into gradient values of scattering points. This process focuses on obtaining stable scatter point distribution information.

#### 3.1.2. Gradient Enhancement and Integral Graph

**Figure 4:**
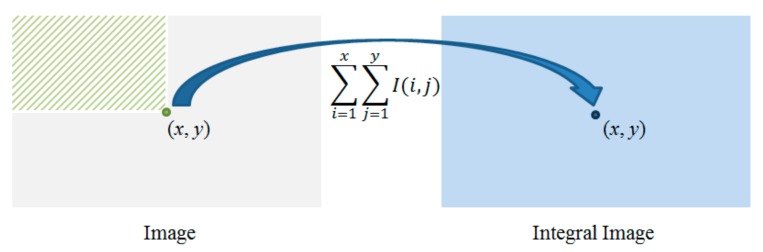
Schematic diagram of integral image.

Next, a gradient integral map is generated based on the gradient feature map for enhancing the gradient information of the around range. The concept of the integral graph was first proposed by Paul Viola et al. [31]. It is applied in a real-time target detection framework. Although the integral graph can also be understood as a graph, the value of any point (*x*, *y*) in the graph refers to the sum of the grayscale values of pixels within the rectangle area from the upper left corner to the current point. The following diagram illustrates the concept of the integral graph and the generated method, as shown in Figure 4.

(15) $$I_{\mathrm{integral}}(x,y)=\sum_{i=1}^{i\leq x}\sum_{j=1}^{j\leq y}I(i,j)$$

If each point of the integral image is calculated according to Equation (15), there is repeated calculation. The integral image is actually an accumulated operation, so it can be optimized by an iteration operation as shown below.

(16) $$I_{\mathrm{integral}}(i,j)=I_{\mathrm{integral}}(i-1,j)+I_{\mathrm{integral}}(i,j-1)-I_{\mathrm{integral}}(i-1,j-1)$$

**Figure 5:**
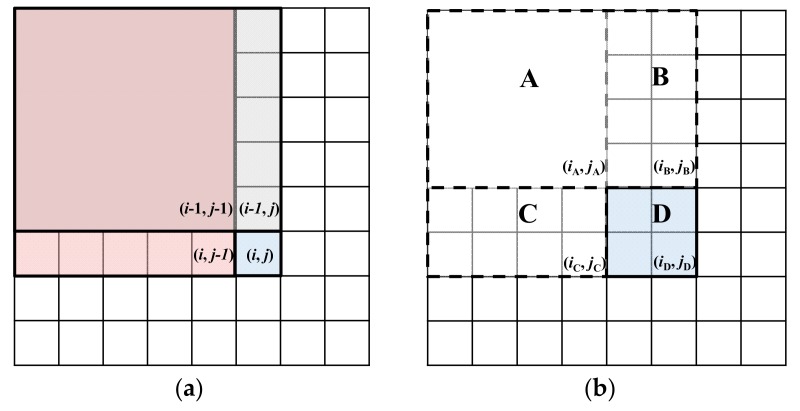
Diagram of integral image fast calculation. (**a**) Entire integral image calculation diagram; (**b**) Sub area-based integral calculation diagram.

In addition, the integral image can be regarded as a look-up table. When the integral image of the sub-area needs to be calculated, the result can be quickly obtained through four angular points by using the following formula, as shown in Figure 5. Moreover, this method also provides great convenience in subsequent Haar feature calculations.

(17) $$\mathrm{sum}(D)=I_{\mathrm{integral}}(i_{D},j_{D})-I_{\mathrm{integral}}(i_{C},j_{C})-I_{\mathrm{integral}}(i_{B},j_{B})+I_{\mathrm{integral}}(i_{A},j_{A})$$

#### 3.1.3. Candidate Areas Extraction

**Figure 6:**
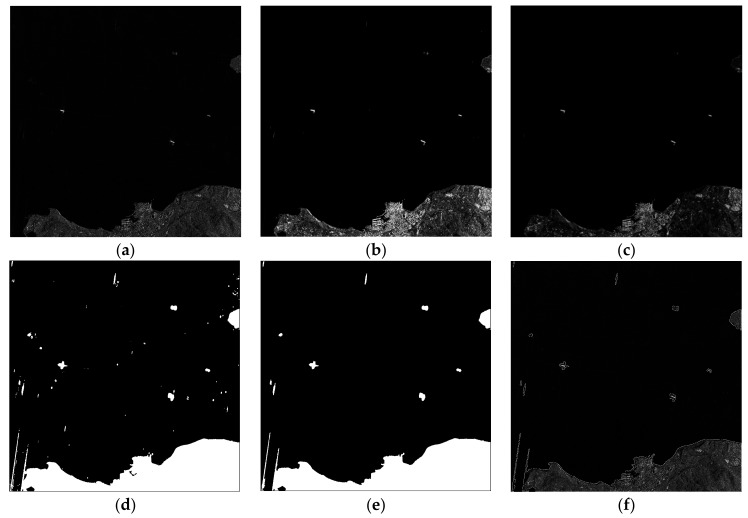
Processing schematic of candidate area extraction. (**a**) Filtered image; (**b**) Gradient image; (**c**) Gradient enhanced graph; (**d**) Adaptive threshold segmentation result; (**e**) Morphological treatment results; (**f**) Marked results on original image.

The gradient integral image is used to enhance the gradient of a region. Select a sub-window with 9 × 9 pixels size, and slide this sub-window overlapping on the gradient image to calculate the sum of the pixels using Equation (17). The result is shown in Figure 6c.

Next, the adaptive segmentation threshold is calculated by the minimum error method. First, we assume the arbitrary gray level threshold *T* is separated the pixels into the target area and background area. Then, calculate the mean and variance of each area:

(18) $$P_{i}(T)=\sum_{g=a}^{b}h{\left(g\right)}_{i},i=1,2$$

(19) μi(T)=[∑g=abh(g)g]Pi(T),i=1,2

(20) σi2(T)=[∑g=ab{g−μi(T)}2⋅h(g)]Pi(T),i=1,2

where *h(g)* is the probability density function with gray level *g*, and *a*, *b* are the gray value range of the background or target. When the parameter *i* = 1, the range values are set as *a* = 0 and *b* = *T*. When the parameter *i* = 2, the range value *a* = *T* + 1 and *b* is set as the maximum gray level.

Second, the objective function of minimum error is obtained as below, according to the idea of minimum classification error [32]:

(21) $$J(T)=P_{1}(T)\cdot \ln\frac{\sigma_{1}^{2}(T)}{{\left[P_{1}(T)\right]}^{2}}+P_{2}(T)\cdot \ln\frac{\sigma_{2}^{2}(T)}{{\left[P_{2}(T)\right]}^{2}}$$

Then, minimize the objective function and get the optimal solution as shown below:

(22) $$\begin{array}{ll} T^{*} & =\arg\left\{\underset{T}{\min}J(T)\right\}\\ & =1+2\left[P_{1}(T)\cdot \ln\sigma_{1}(T)+P_{2}(T)\cdot \ln\sigma_{2}(T)\right]-2\left[P_{1}(T)\cdot \ln P_{1}(T)+P_{2}(T)\cdot \ln P_{2}(T)\right] \end{array}$$

After that, the gradient enhanced image is binarized with the obtained threshold, and then some morphological operations are performed, such as hole filling. The result is shown in Figure 6d.

The segmentation results are the areas with abundant texture information, mainly including land, islands, ships and sea clutters. However, these different targets have a variant size. Thus, we can make a general screening through the area of a connected region. For example, the area of false alarm in the background is much smaller than that of the targets. Therefore, we can delete the area through a threshold of the total pixel number. The threshold is set under the area of target connected domain. In this paper, we select approximately 200 as the threshold. The result is shown in Figure 6e. Further, land or island areas are much larger than ships, so a similar approach can be used to remove the land area from the target candidate area with a relatively safe threshold.

### 3.2. Ship Target Identification

After obtaining the candidate areas, it is necessary to identify the ship targets from these areas. In this section, we will present an optimized Haar-like method to extract the ship features in SAR imagery. Finally, the features are classified by Adaboost to distinguish the ship targets from the candidate areas.

#### 3.2.1. Haar-Like Feature Optimized

**Figure 7:**
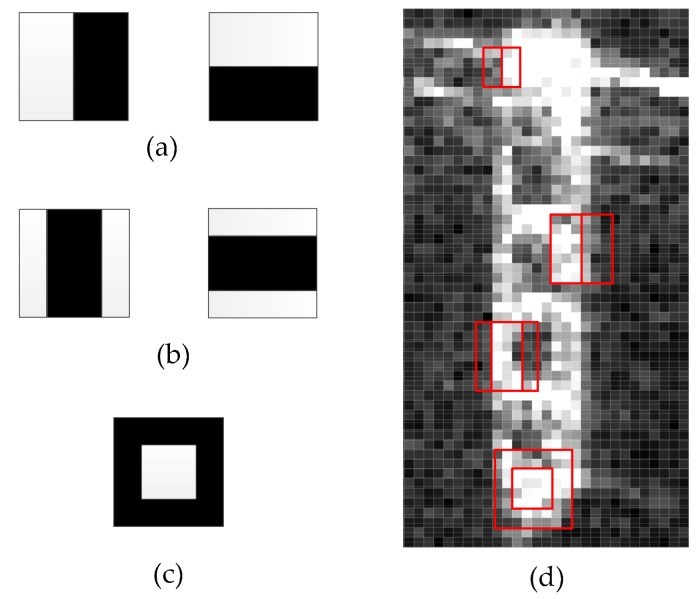
Schematic representation of Haar-like features. (**a**) Edge feature template; (**b**) Line feature template; (**c**) Center feature template; (**d**) A ship sub-image with different feature templates.

The Haar-like feature is one of the common character-describing operators in the field of computer vision, and it has been used in face recognition. It has the characteristics of a flexible template, variable scale and low computational complexity. There are three main types, including the edge feature, line feature and the center, as shown in Figure 7. Each feature template has two kinds of rectangles, white rectangles and black rectangles. Additionally, we define the feature value of the template as the difference between the sum of white rectangular pixels and the sum of black rectangular pixels. The feature value of the Haar-like feature reflects the gray level distribution within the template. Then, by changing the scale and position of the feature template, a set of hierarchical feature sets can be generated.

The ship targets in the SAR image are a collection of strong scattering points whose shape is similar to a slender rectangle. The characterization of Haar-like features well matches the characteristics of the ship targets.

Because of the category, location and size of the Haar-like template are variable, many feature values are generated. We can employ the integral image method to simplify the calculation. First, we generate the integral image of the filtered image. Then, take the integral image as a look-up table, and we can obtain the area of the rectangle by the four points. Thus, it can improve the operating speed and ensure real-time processing.

In practice, the orientation of a ship in the image is arbitrary. But the Haar-like feature templates usually only exhibit horizontal, vertical and 45-degree directions [33]. Thus, these templates cannot describe the ship characters properly. If we add templates in multiple directions, it is difficult to calculate in the presence of the discrete image and submit redundant features to classification.

To solve this problem, we propose a solution with a Radon transform. It is found that the number of strong scattering pixels distributed along the ship direction is greater than that of the others. Thus, we employ the discrete Radon transform method [34] to determine the pixels distribution. The Radon transform maps the pixels distributed along a certain direction into a new point of transformed space. The point intensity distribution within transformed space shows the existence possibility of the ship direction in the original image. After the ship direction is confirmed, rotate the patches and make all the ships distributed in the same direction, for example, the vertical direction.

**Figure 8:**
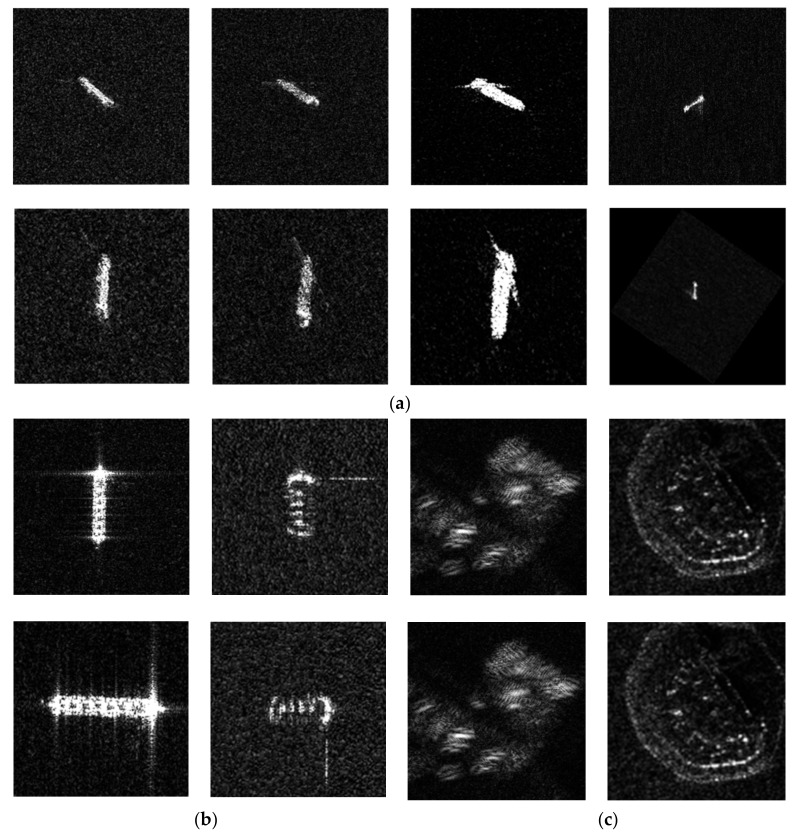
Schematic diagram of a Radon transform, where the upper row is the original patch and the following is the transformed result. (**a**) Ship patches transformed to the vertical direction; (**b**) Ship patches transformed to the horizontal direction; (**c**) Non-ship patches transformation schematic.

A schematic of the Radon transform results is shown in Figure 8a. These results show that most ship slices can be rotated to the desired angle, even the small ships as shown in the last column, which appear the rectangular shape in image. However, in Figure 8b, some patches are rotated to the horizontal angle because of the strong scattering point at the ship’s bow influence in the main direction. In addition, there are some non-ship patches rotation results shown in Figure 8c. These results are basically unchanged because non-ship targets do not have an obvious line distribution. In sum, we choose vertical and horizontal feature templates of Haar-like features after being optimized.

We also need to build a training set and extract the features to train a classifier by using the modified Haar-like method. We re-mark the candidate areas into square patches and separate these patches into ship patches and false alarm patches. In order to ensure that each training sample has the same patch size and feature numbers, all the patches are resized to a fixed size. If the patch size is large, the dimension of the Haar-like features increases, which will burden the classifier training. If the patch size is small, the detail information will be lost during down-sampling. Therefore, due to the GF-3 image resolution and the actual size of the ship, we set the training set patch size to 30 × 30 pixels. Besides, we also resize the test samples to 30 × 30 pixels during the target confirmation step.

#### 3.2.2. Target Identification Based on Cascade Classifier

A classifier is needed to remove the false alarm in the candidate regions by using these Haar-like features. However, the number of features extracted by the Haar-like method is very large, nearly in the tens of thousands. Training with traditional classifiers will cause the curse of dimensionality. To solve this problem, the cascade classifier becomes the most effective approach. Thus, we employ the Adaboost classifier to distinguish ships and false alarms in candidate areas. The main purpose of Adaboost is to choose which features are the most effective and to combine these features properly to obtain better identification ability.

AdaBoost is a kind of iterative algorithm, and it aims to train different weak classifiers and assemble these weak classifiers to construct a strong classifier. In each round of training, the weight of each sample is determined based on a classified result and the overall accuracy of the last instance. Then, the new data with modified weights are sent to the next classifier. Finally, the strong classifier mixes the weak classifiers together and makes a final decision.

The AdaBoost method has the following characteristics. First, it does not need to concern feature selection, and the weights of each feature are adaptively updated during iteration. Second, it does not need to be concerned with overfitting due to the amounts of features. Finally, the structure of the weak classifier is extremely simple. Although the accuracy of a single weak classifier is low, a high-precision classifier can be obtained by concatenating multiple weak classifiers. Suppose there is a *K*-level cascade classifier; *f_i_* represents the error rate of the *i*-th classifier, and *d_i_* represents the detection rate of the *i*-th classifier. The detection rate *D* can be expressed as:

(23) $$D=\overset{K}{\underset{i=1}{\Pi }}d_{i}$$

The error rate *F* can be expressed as:

(24) $$F=\overset{K}{\underset{i=1}{\Pi }}f_{i}$$

For example, suppose there is a classifier with twenty layers. The detection rate is *d_i_* = 0.995 per layer, and the error rate is *f_i_* = 0.5. Then, the detection rate is D ≈ 0.9 after the final concatenation, and the error rate is *F* ≈ 5 × 10^−7^.

According to the above section, the Haar-like features are extracted from patches containing the ship to form a positive sample set. Additionally, the Haar-like features are extracted from the non-ship patches, containing reefs and sea clutter, to form a negative sample set. The positive and negative samples are merged into a set ***X*** = (*x_1_*, *x_2_*, …, *x_n_*), where *n* is the total number of training samples, and each sample *x_i_* corresponds to a label *y_i_* ∈ {1, −1} representing the ship patch or the non-ship patch.

Suppose that *j* represents the number of training iterations, and ***W_j_*** = (*w_j1_*, *w_j2_*, …, *w_jn_*) represents the weight value of each sample at the *j*-th iteration. Additionally, set the initial weight of each sample equal to 1/*n*. Assume that classifier *h* defines the error classification rate *e_j_* for the weighted samples as:

(25) $$e(h)=\sum_{i=1}^{n}\frac{\omega_{j,i}}{2}(1-h(x_{i})y_{i})$$

The above formula shows that the error rate of *h* of the training set is the sum of the weights of misclassified samples.

When *j* = 1, 2, …, *T*, the following computations are repeated.

First, choose the weak learner to learn which has the smallest classified error according to the weight of the current sample:

(26) $$h_{j}=\arg\underset{h}{\min}\;e(h)$$

The weight of this weak classifier *h_j_* is defined by:

(27) $$\alpha_{j}=\frac{1}{2}\ln\frac{1-e(h_{j})}{e(h_{j})}$$

As can be seen from above equation, when *e*(*h_j_*) ≤ 0.5, then *α_j_* ≥ 0, and *α_j_* increases along with the decrease in *e*(*h_j_*). This means that the weak classifier with smaller classification error plays a more important role in the final classifier.

Finally, update the weight of the training data:

(28) $$\omega_{j+1,i}=\frac{\omega_{j,i}}{Z_{j}}\exp(-\alpha_{j}y_{i}h_{j}(x_{i})),\forall i=1,2,\ldots ,n$$

where *Z_j_* is a normalization factor, expressed as:

(29) $$Z_{j}=\sum_{i=1}^{n}\omega_{j,i}\exp(-\alpha_{j}y_{i}h_{j}(x_{i}))$$

**Figure 9:**
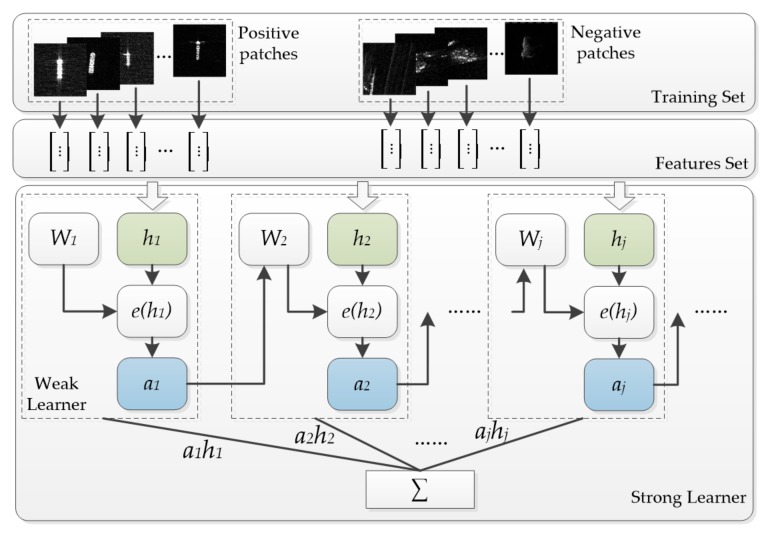
Adaboost training process diagram.

From the above description, we find that the Adaboost training process is a continuous cognitive process on the wrongly classified samples. Moreover, in the process of training, the weights of samples are updated continuously according to the classification results. For the correctly classified samples, because the classifiers have recognition ability, their weights are reduced. On the contrary, for the samples with wrong classification, their weights should be increased to improve cognition. After iteration processing, the weak classifier achieves optimization. The training process is shown in Figure 9. Finally, the optimized weak classifiers generate a strong classifier, expressed as:

(30) $$H(x)=sign(\sum_{j=1}^{T}\left(\alpha_{j}h_{j}(x)\right)$$

**Figure 10:**
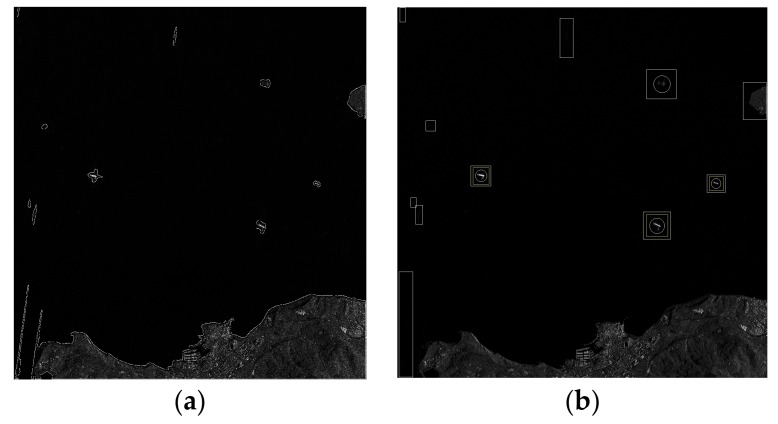
Result of target identification. (**a**) The candidate areas; (**b**) Detection result labelled on the image, where the white rectangle indicates the candidate area, and the yellow rectangle indicates the final detection results, and the white circle indicates the ground truth.

After the classifier is trained by the training set, the candidate areas are resized and identified through Adaboost. As a result, the ship target areas are retained and the false alarms are removed, as show in Figure 10.

## 4. Experiments and Results

In this section, a number of experiments are designed, and evaluation methods are presented. All of the experiment data were gathered using the Gaofen-3 SAR satellite C band. GF-3 has many imaging modes and we mainly use single-polarization imagery from three kinds of imaging modes listed as Table 1. These images are acquired in November 2016 with the coverage of South and East China Sea. There are a total of 40 scenes with approximately 13,000 × 14,000 pixels in size, including three scenes of 1 m resolution, 21 scenes of 3 m resolution and 16 scenes of 5 m resolution. These scenes contain coastal ports and ship targets on the sea. We cut out nearly 400 patches form the training set, including the ship target and various false alarms, such as the island and sea clutters. In addition, beyond the training set, we select 12 images of GF-3 as test data sets, which contain a variety of typical scenarios and different sea conditions.

### 4.1. Experiment of Noise Reduction 

In this section, we design the experiment to obtain the optimized parameter of de-noise filter, and then explain the adaptive characteristic of the filter with different type of patches. Above all, we employ some quantitative evaluation indexes to appraise the employed adaptive speckle reduction method. We select the equivalent number of looks (ENL) and structural similarity index (SSIM) [36] to evaluate the quantity performance. The ENL is the index for describing the relative intensity of speckle and the SSIM is the index for describing the texture preserving effect. The formulas are shown below:

(31) $$M_{\mathrm{ENL}}=\frac{\mu^{2}}{\sigma^{2}}$$

(32) $$\begin{array}{l} \mathrm{SSIM}(X,Y)=\frac{\left(2\mu_{X}\mu_{Y}+C1)(2\sigma_{XY}+C2\right)}{\left(\mu_{X}^{2}+\mu_{Y}^{2}+C1)(\sigma_{X}^{2}+\sigma_{Y}^{2}+C2\right)}\\ C1={\left(0.01\times L\right)}^{2},C2={\left(0.03\times L\right)}^{2} \end{array}$$

where *X* and *Y* indicate the original and processed images, *μ* and *σ*^2^ are the mean and variance within the image, and *L* is the pixel dynamic range. In this paper, we select the *L* = 256 because each pixel is expressed by 8 bits.

**Figure 11:**
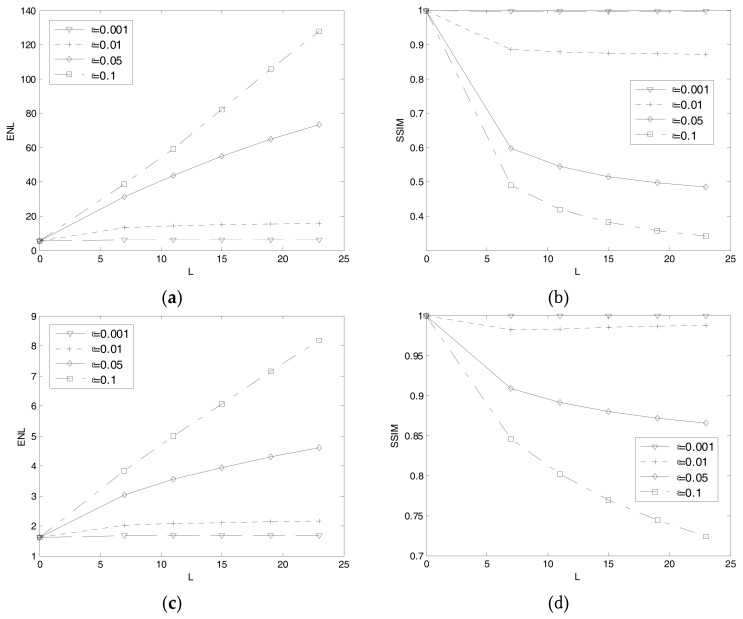
The index changing curve with different parameters. (**a**) Result of ENL changing curve in homogeneous region; (**b**) Result of SSIM changing curve in homogeneous region; (**c**) Result of ENL changing curve in target region; (**d**) Result of SSIM changing curve in target region.

Then, we optimize the parameters *r* and *ε* in the filter. We select the homogeneous region and target region respectively to verify the filter performance under different parameters. We change the values of parameters *l* and *ε* for a relevant sample of values to obtain the changing curve, as shown in Figure 11. The experiment result shows the sensitivity of the parameters. First, the increase of ENL is accompanied by the decrease of SSIM. When the SSIM is less than 0.9, the target texture information will be fuzzy and the detection result will be impacted. Second, the curves of different scenes have different amplitudes but have the same variation tendency, which illustrates the adaptive character of the filter. Third, when *ε* is too low, the filtering effect is not significant. When *ε* is too high, the target texture is fuzzed up and the SSIM value is under 0.5. So the choice of parameter *ε* must guarantee the texture information preserved. Besides, we notice that the performance of filter is invariant when *l* is greater than a certain value. So we set optimize value of *l* as the inflection point. To sum up, we set the optimize value as *ε* = 0.05 and *l* = 7 and the filter has the best performance of de-noise and texture maintain.

**Figure 12:**
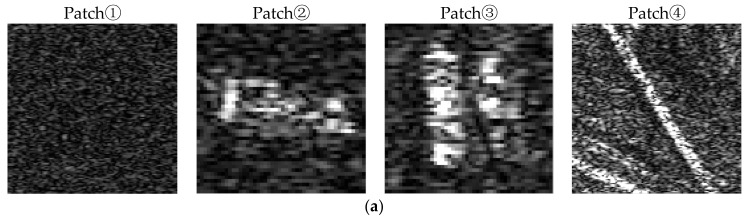

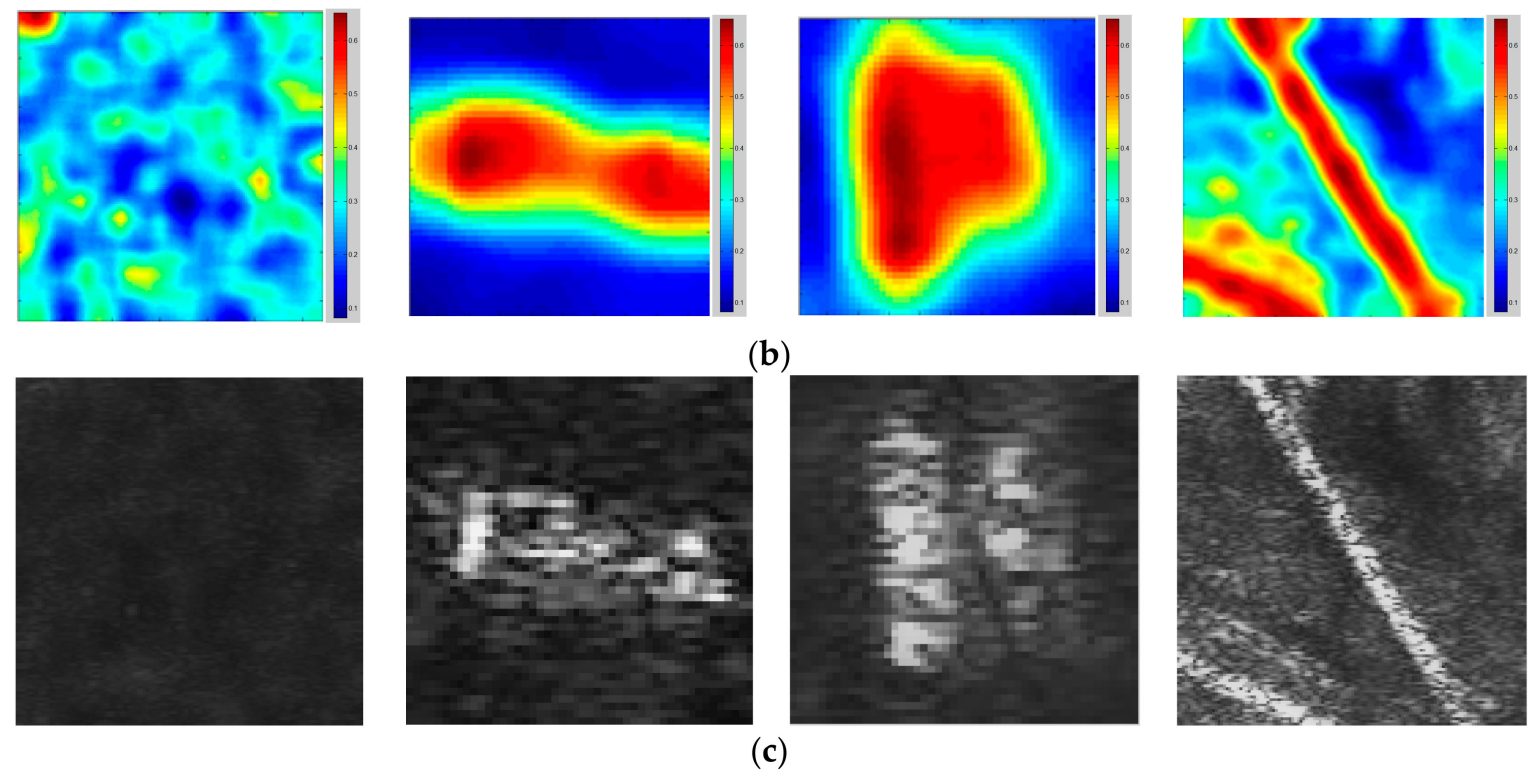
Schematic diagram of filtered effect: (**a**) Original patches; (**b**) Graphics of filtered maintain parameter *a*; (**c**) Result of filtering.

In order to illustrate the performance of the filter, a set of typical targets patches and the filtering results are shown in Figure 12. The corresponding quantity performances are listed in Table 2, where the mean and variance are expressed in normalization. Among them, patch 1 contains a flat sea surface. Thus, the filtered maintain parameters *a* are mostly low, and the effect of smoothing is obvious. Thus, the index of ENL has significantly improved after filtering. Patch 2 contains a single ship, patch 3 contains double ships, and patch 4 contains a straight artificial target in the land area. From Figure 9b, the filtered maintain parameters within the target position are obviously higher than those in the background, and appear to have the same outlines as the targets. Thus, in the filtered result, the background pixels are smoothed and the target information can be retained effectively.

**Figure 13:**
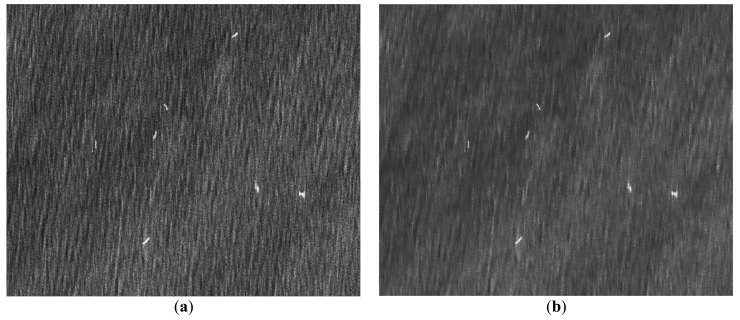
Schematic diagram of filtered effect in bad sea condition under huge waves: (**a**) Original image; (**b**) Filtered image.

Figure 13a is a part of an SAR image of bad sea condition under huge waves. It can be seen that the pixel intensity in the background is relatively high, which has a serious impact on target detection. Figure 13b shows the filtered image. From this image, the background noise is apparently suppressed. Thus, the proposed filtering algorithm can also smooth the background noise at the same time and provide a clear image for target detection.

### 4.2. Experiment of Detection Method

#### 4.2.1. Key Parameters Analysis of Haar-Like Feature Extraction

Haar-like feature extraction plays an important role in ship target identification. However, Haar-like features have multiple templates and their sizes are optional. Therefore, in this section, the most effective feature template for ship identification is selected.

The first step is to select the effective Haar-like feature template. Thus, we picked several pairs of patches from positive or negative samples after the Radon transform, as shown in Table 3. The first group shows the typical situation of similar ship targets. The second group shows the patches of different kinds of ships. The third group shows the false alarm patches. The fourth group shows the patches of the ship target and false alarm. The edge feature template, the line feature template and the center feature template are used to extract the feature values of the two patches in each group. In order to obtain the detail feature description, we use the templates with the size of 4 × 4 pixels. Then, calculate the correlation coefficient of the feature values in each group as below:

(33) $$\rho (P_{1},P_{2})=\frac{1}{N-1}\sum_{i=1}^{N}\left(\frac{P_{1i}-\mu_{p_{1}}}{\sigma_{p_{1}}}\right)\left(\frac{P_{2i}-\mu_{p_{2}}}{\sigma_{p_{2}}}\right)$$

where *μ* and *σ* are the mean and standard deviation of feature values, and *N* is the total feature number of the patch, and *P_i_* indicates the *i*-th feature value. The correlation coefficients of each group are shown in Table 3.

It can be seen from the results that the distinguishing ability of the edge and line feature templates is obviously better than that of the center template. Therefore, we use the edge and line feature templates.

In the second step, select the property size of the template. Because the patch size is 30 × 30 pixels, we choose the template size as 4 × 4, 6 × 6, 8 × 8, 10 × 10 and 12 × 12 pixels, and also combine these templates together successively. In order to evaluate the quantitative performance, indices of target detection are defined as below:

(34) $$\mathrm{Recall}=\frac{N_{d}}{N_{g}}$$

(35) $$\mathrm{Precision}=\frac{N_{d}}{N_{d}+N_{f}}$$

(36) $$\mathrm{FoM}={\left(\frac{1}{\mathrm{Precision}}+\frac{1}{\mathrm{Recall}}-1\right)}^{-1}=\frac{N_{d}}{N_{f}+N_{g}}$$

where *N_d_* is the number of detected targets, *N_f_* is the number of false alarms, and *N_g_* indicates the number of ground truths.

**Figure 14:**
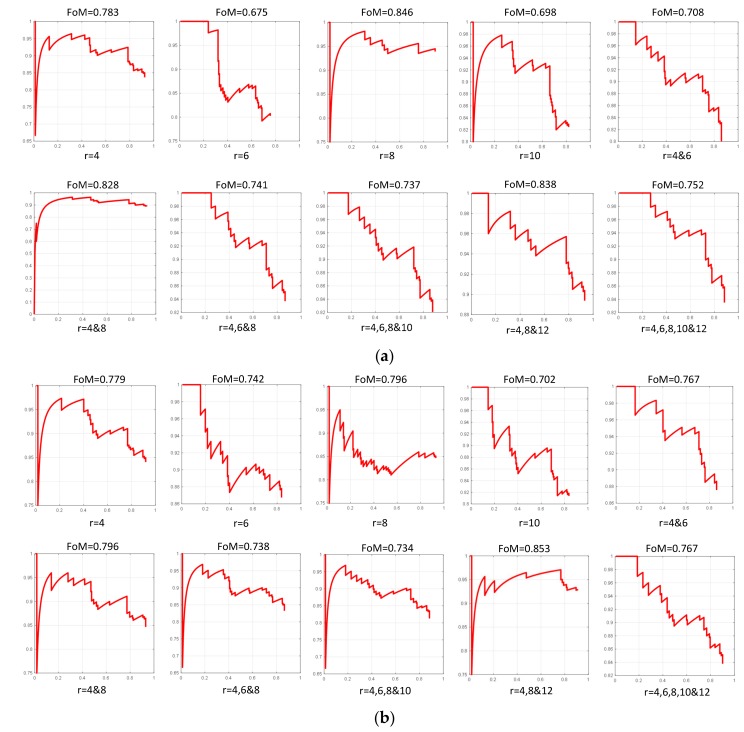

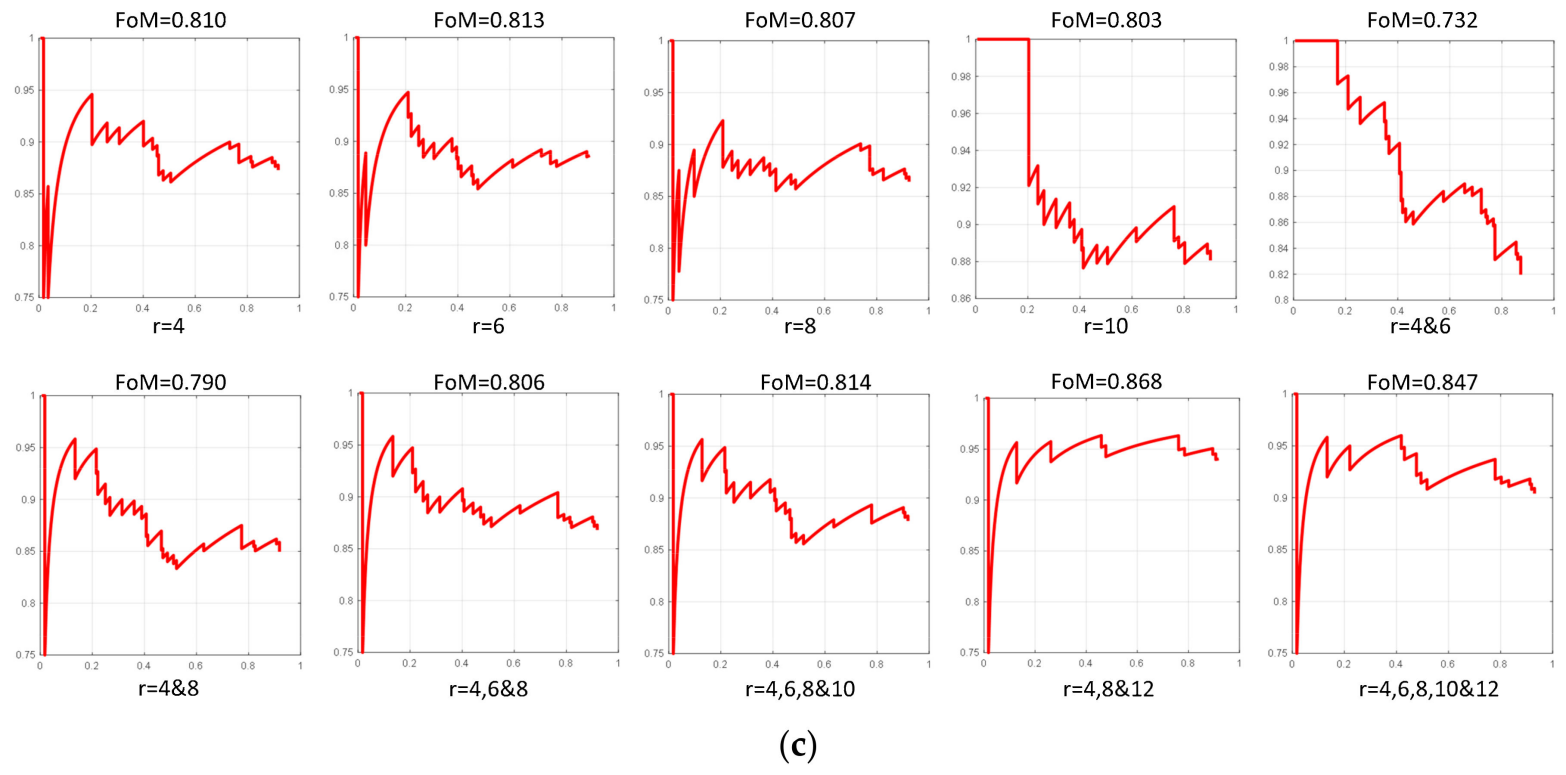
Precision-recall curve of different template sizes. The x axis indicates recall and the y axis indicates precision. (**a**) Results of different sizes in line feature templates; (**b**) Results of different sizes in edge feature templates; (**c**) Results of different sizes in edge feature and line feature combined templates.

We use the line feature template with these different sizes to train the classifier and experiment on the testing data. The detection results are shown in Figure 14a. In the same way, the results of the edge feature template and line-edge combined template are shown in Figure 14b,c.

In sum, from the experiments, it is found that the edge feature performs better than the line feature. However, the edge and line features combined can achieve improvements over the use of a single feature. Furthermore, the template size grouped by 4 × 4, 8 × 8, and 12 × 12 pixels has the best performance. Thus, we select the final templates in the Haar-like method, which is the edge feature and line feature in the sizes 4 × 4, 8 × 8 and 12 × 12 pixels, respectively.

#### 4.2.2. Key Parameters Analysis of Adaboost Classifier

**Figure 15:**
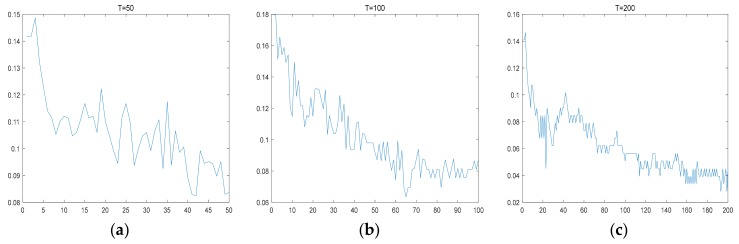

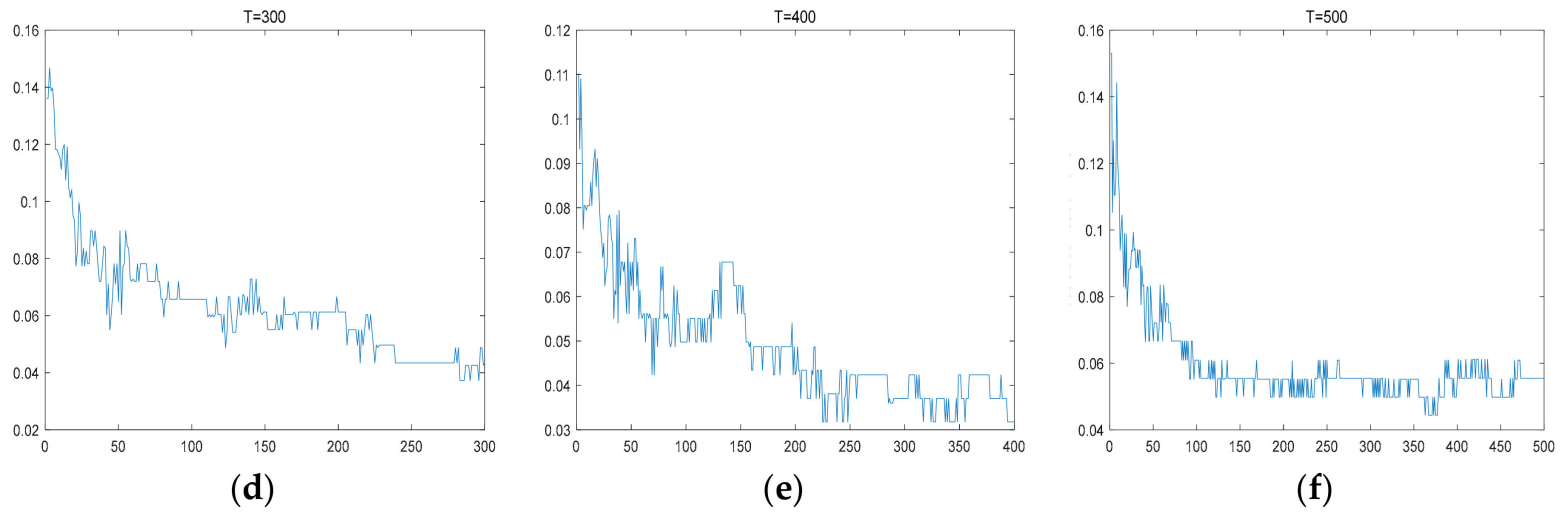
Classification error curve of different cascading layers. The *x* axis indicates the cascading layer and the *y* axis indicates the classification error. (**a**) With 50 cascading layers; (**b**) With 100 cascading layers; (**c**) With 200 cascading layers; (**d**) With 300 cascading layers; (**e**) With 400 cascading layers; (**f**) With 500 cascading layers.4.3. Detection Result Analysis.

In the proposed method, the cascading layer of Adaboost is also an important parameter. We use the decided Haar-like features and fixed testing set to train the Adaboost classifier through different cascading layers. The experiment results are shown in Figure 15.

From the experiment results, it can be seen that the classification error rate gradually converges with the number of iteration layers. When the cascading layer reaches approximately 200–300, the classification error achieves the minimum value. When the cascading layer exceeds 300, the performance of the classifier is basically the same.

**Figure 16:**
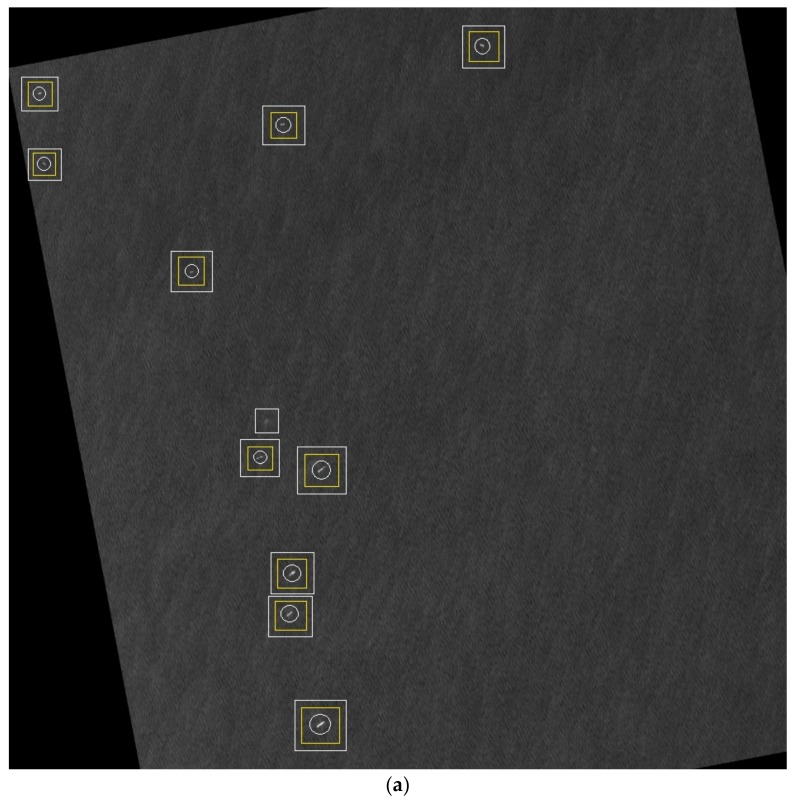

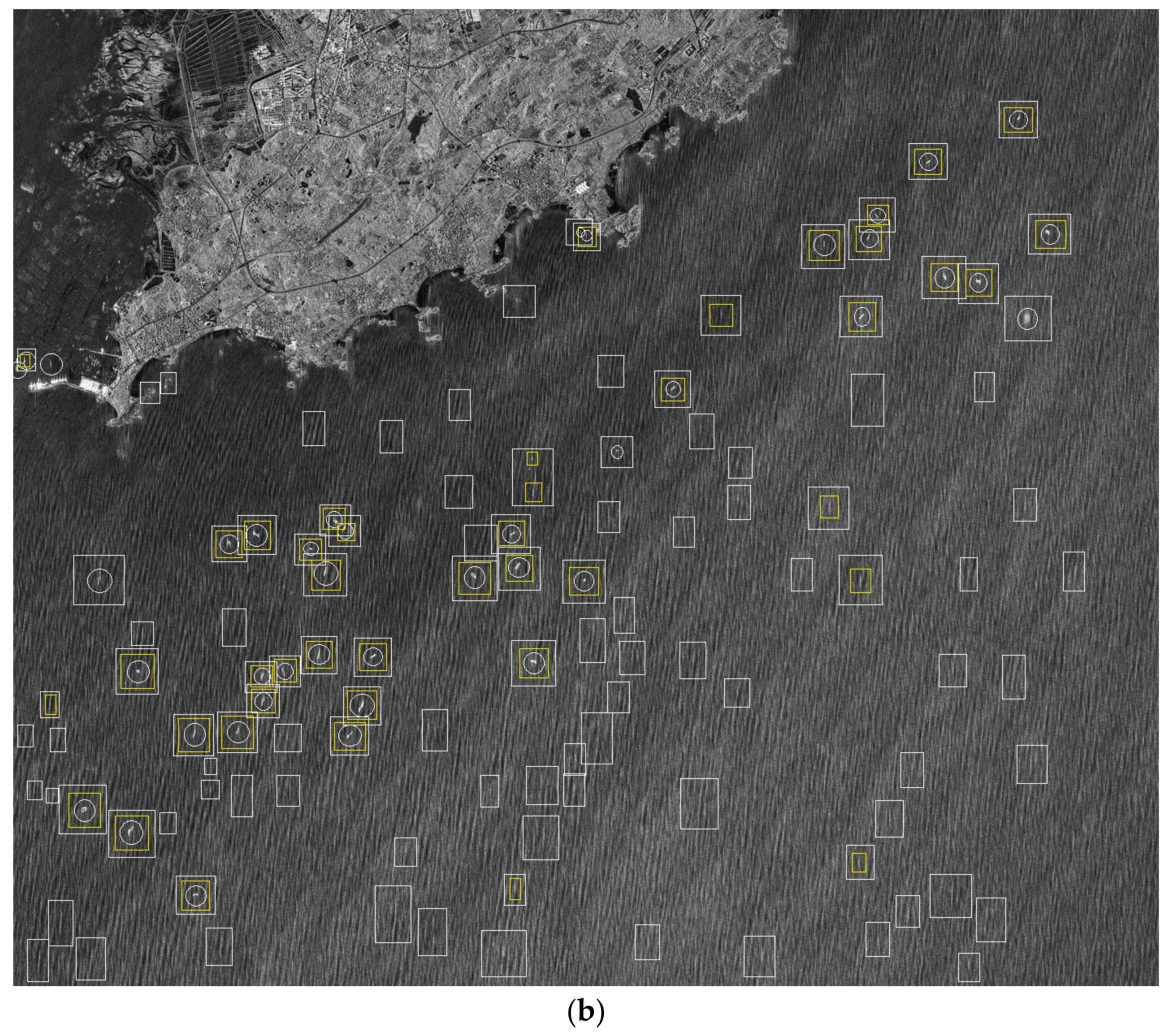
Detection result of the proposed method. The white rectangle indicates the candidate area extracted by the proposed algorithm. The yellow rectangle indicates the final detection results. The white circle indicates the ground truth. (**a**) Detection result in clean sea surface; (**b**) Detection result in clutter sea surface.

After the parameters are confirmed, the typical detection results of the proposed method in large-scale SAR images are presented in Figure 16. In particular, Figure 16b shows the detection result in bad sea condition under huge waves. It is difficult to detect the ship target using an unsupervised approach with just a few false alarms.

To demonstrate the advantage of the proposed method, we employ other methods represented in [20,37,38] for the sake of comparison. Table 4 lists the quantitative comparisons. To ensure the fairness of the experiment, the same database was used for other methods. In addition, the parameters of the contrastive methods were adjusted to the optimal state.

From the experiment, we can see that the proposed method performs better than typical algorithms, in particular in bad sea condition under huge waves. The preprocessing of the adaptive filter removed the speckle noise and clutters without decreasing the target. Then, the gradient information enhancement effectively improved segmentation accuracy in the SAR image. Finally, the modified Haar-like feature extraction method describes the ship target characters more accurately and conveniently. However, some targets are missing in the detection results, because these targets are defocused when moving. So, their shapes are changed, which is not included in the training set.

The proposed method entails less computing time also. We experiment on the Matlab platform at a personal computer equipped with an Inter i5-4200M 2.50-GHz processor and 8 GB of RAM. We selected 10 scenes from the testing sets and calculated the mean of the run time. The time consumption results are shown in Table 4, in which the comparison results are provided by the authors of [20]. The standard CFAR takes approximately 710 s to process one scene image. The calculating speeds of algorithms [20,37,38] are almost 2–5 times as long as the standard CFAR. However, the proposed method spends approximately 180 s to finish the target detection, including the filtering processing.

In addition, we map the proposed method to Xinlinx Virtex-5 FPGA processor. We use the XC5VFX130T FPGA with 150 MHz clock frequency and use the DDR2 as external storage device. This FPGA logical resources can contain three groups of the proposed method pipelined parallel processing. It only takes less than 3 s per scene to finish the ship detection. First, the proposed method is composed of linear operations, which are computed rapidly and are easy to map in the embedded system. Second, the integral image is employed in our method to accelerate the gradient enhance step and Haar-like feature calculation. Finally, the modified Haar-like feature extraction method reduced the number of templates and released the computation burden. In sum, the proposed method has high detection accuracy and high real-time performance.

## 5. Conclusions

In this paper, a gradient integral feature based ship detection method for SAR imagery is proposed. In the preprocessing step, a kind of adaptive filter is employed to reduce the speckle noise and background clutter. We employ a sliding window to filter the whole image. The flat area will be smoothed and the textured area will be preserved. In the candidate area extraction step, a sea-land segmentation method is proposed based on gradient integral enhancement. This method can segment the offshore land area accurately and extract the candidate areas of the ship target effectively. In addition, the integral image method is employed to accelerate the computation. In the target identification step, a feature extraction strategy is proposed based on a Haar-like method and Radon transform. This strategy solved the problem of ship orientation variety. The Radon transform is used for rotating the ship patches within a unified direction. Then, the number of Haar-like templates is reduced. Experiments on large-scale SAR images from a GF-3 satellite verify the proposed method is effective and robust when applied in bad sea condition under huge waves. In the future, we are going to increase the training samples within varied situations, such as the defocused ship. The proposed method also has the potential for on-board processing and support shipping management.

## Figures and Tables

**Table 1:** GF-3 main technical specifications of different imaging modes used [35].

Imaging Mode	Spatial Resolution (m)			Nominal Width (km)	Polarization Mode
	Nominal	Azimuth	Range		
Spotlight	1	1.0–1.5	0.9–2.5	10 × 10	optional single-pol
Ultra-fine stripmap	3	3	2.5–5	30	optional single-pol
Fine stripmap 1	5	5	4–6	50	optional dual-pol

**Table 2:** Quantitative comparison with filtering effect

Imaging	Original			Filtered		
	Mean	Var	ENL	Mean	Var	ENL
Patch 1	0.153	0.074	4.228	0.155	0.012	163.375
Patch 2	0.218	0.176	2.570	0.219	0.158	2.365
Patch 3	0.645	0.280	5.302	0.584	0.182	10.271
Patch 4	0.563	0.303	3.460	0.526	0.195	7.254

**Table 3:** Experiment of different feature template.

No.	Patch 1	Patch 2	Correlation Coefficients		
			Edge	Line	Center
1	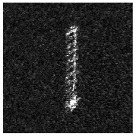	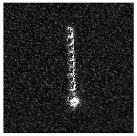	0.9723	0.9554	0.9910
2	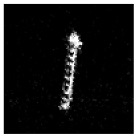	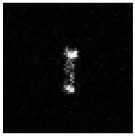	0.8326	0.7530	0.7694
3	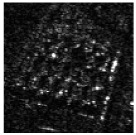	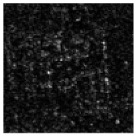	0.4728	0.2898	0.9647
4	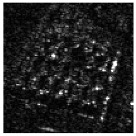	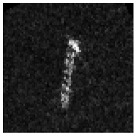	0.0446	0.0725	0.9158

**Table 4:** Detection results of different methods.

Method	Precision	Recall	FoM	Time (s)
Standard CFAR	0.7400	0.683	0.5508	716
Bilateral CFAR [37]	0.8739	0.818	0.7316	1622
Feature Analysis [38]	0.8211	0.780	0.6667	3427
IS domain CFAR [20]	0.8721	0.832	0.7415	2938
Proposed	0.9405	0.9186	0.8681	181

## References

[1] El-Darymli K., Gill E.W., McGuire P., Power D., Moloney C. (2016). Automatic target recognition in synthetic aperture radar imagery: A state-of-the-art review. IEEE Access.

[2] Liu S., Cao Z., Wu H., Pi Y., Yang H. (2016). Target detection in complex scene of SAR image based on existence probability. EURASIP J. Adv. Signal Process..

[3] Song S., Xu B., Li Z., Yang J. (2016). Ship Detection in SAR Imagery via Variational Bayesian Inference. IEEE Geosci. Remote Sens. Lett..

[4] Tao D., Doulgeris A.P., Brekke C. (2016). A segmentation-based CFAR detection algorithm using truncated statistics. IEEE Trans. Geosci. Remote Sens..

[5] Yang M., Zhang G. (2016). A novel ship detection method for SAR images based on nonlinear diffusion filtering and Gaussian curvature. Remote Sens. Lett..

[6] Novak L.M., Owirka G.J., Netishen C.M. (1993). Performance of a high-resolution polarimetric SAR automatic target recognition system. Linc. Lab. J..

[7] Goldstein G.B. (1973). False-alarm regulation in log-normal and Weibull clutter. IEEE Trans. Aerosp. Electron. Syst..

[8] Li H.C., Hong W., Wu Y.R., Fan P.Z. (2011). On the empirical-statistical modeling of SAR images with generalized gamma distribution. IEEE J. Sel. Top. Sign. Proces..

[9] Di Bisceglie M., Galdi C. (2005). CFAR detection of extended objects in high-resolution SAR images. IEEE Trans. Geosci. Remote Sens..

[10] Kuttikkad S., Chellappa R. Non-Gaussian CFAR techniques for target detection in high resolution SAR images. Proceedings of the IEEE International Conference on Image Processing (ICIP 1994).

[11] Leng X., Ji K., Zhou S., Xing X., Zou H. (2016). An adaptive ship detection scheme for spaceborne SAR imagery. Sensors.

[12] Rey M.T., Drosopoulos A., Petrovic D. (1996). A Search Procedure for Ships in RADARSAT Imagery.

[13] Crisp D.J. (2004). The State-of-the-Art in Ship Detection in Synthetic Aperture Radar Imagery.

[14] Qin X., Zhou S., Zou H., Gao G. (2013). A CFAR detection algorithm for generalized gamma distributed background in high-resolution SAR images. IEEE Geosci. Remote Sens. Lett..

[15] Gao G., Ouyang K., Luo Y., Liang S., Zhou S. (2017). Scheme of Parameter Estimation for Generalized Gamma Distribution and Its Application to Ship Detection in SAR Images. IEEE Trans. Geosci. Remote Sens..

[16] El-Darymli K., McGuire P., Power D., Moloney C. (2013). Target detection in synthetic aperture radar imagery: A state-of-the-art survey. J. Appl. Remote Sens..

[17] Gao G. (2011). A parzen-window-kernel-based CFAR algorithm for ship detection in SAR images. IEEE Geosci. Remote Sens. Lett..

[18] Lang H., Zhang J., Wang Y., Zhang X., Meng J. (2016). A synthetic aperture radar sea background distribution estimation by n-order Bézier curve and its application in ship detection. Acta Oceanol. Sin..

[19] Tian S.R., Wang C., Zhang H. An improved nonparametric CFAR method for ship detection in single polarization synthetic aperetuer radar imagery. Proceedings of the IEEE Geoscience and Remote Sensing Symposium (IGARSS 2016).

[20] Wang C., Bi F., Zhang W., Chen L. (2017). An Intensity-Space Domain CFAR Method for Ship Detection in HR SAR Images. IEEE Geosci. Remote Sens. Lett..

[21] Dai H., Du L., Wang Y., Wang Z. (2016). A modified CFAR algorithm based on object proposals for ship target detection in SAR images. IEEE Geosci. Remote Sens. Lett..

[22] Zhai L., Li Y., Su Y. (2016). Inshore Ship Detection via Saliency and Context Information in High-Resolution SAR Images. IEEE Geosci. Remote Sens. Lett..

[23] Wang S., Wang M., Yang S., Jiao L. (2017). New hierarchical saliency filtering for fast ship detection in high-resolution SAR images. IEEE Trans. Geosci. Remote Sens..

[24] Wang X., Chen C. (2016). Adaptive ship detection in SAR images using variance WIE-based method. Signal Image Video Process..

[25] Wang X., Chen C. (2017). Ship detection for complex background SAR images based on a multiscale variance weighted image entropy method. IEEE Geosci. Remote Sens. Lett..

[26] Bentes C., Frost A., Velotto D., Tings B. Ship-iceberg discrimination with convolutional neural networks in high resolution SAR images. Proceedings of the 11th European Conference on Synthetic Aperture Radar (EUSAR 2016).

[27] Schwegmann C.P., Kleynhans W., Salmon B.P., Mdakane L.W., Meyer R.G. Very deep learning for ship discrimination in synthetic aperture radar imagery. Proceedings of the IEEE Geoscience and Remote Sensing Symposium (IGARSS 2016).

[28] Kang M., Leng X., Lin Z., Ji K. A modified faster R-CNN based on CFAR algorithm for SAR ship detection. Proceedings of the IEEE 2017 International Workshop on Remote Sensing with Intelligent Processing (RSIP 2017).

[29] Massonnet D., Souyris J.C. (2008). Imaging with Synthetic Aperture Radar.

[30] Hao S., Liang C., Yin Z., Jian Y., Zhu Y. A Novel Method of Speckle Reduction and Enhancement for SAR Image. Proceedings of the IEEE Geoscience and Remote Sensing Symposium (IGARSS 2017).

[31] Viola P., Jones M. Rapid object detection using a boosted cascade of simple features. Proceedings of the 2001 IEEE Computer Society Conference on Computer Vision and Pattern Recognition (CVPR 2001).

[32] Kittler J., Illingworth J. (1986). Minimum error thresholding. Pattern Recognit..

[33] Lienhart R., Maydt J. An extended set of haar-like features for rapid object detection. Proceedings of the 2002 IEEE International Conference on Image Processing (ICIP 2002).

[34] Beylkin G. (1987). Discrete radon transform. IEEE Trans. Acoust. Speech Signal Process..

[35] Zhang Q.J. (2017). System design and key technologies of the GF-3 satellite. Acta Geod. Cartogr. Sin..

[36] Wang Z., Bovik A.C., Sheikh H.R., Simoncelli E.P. (2004). Image quality assessment: From error visibility to structural similarity. IEEE Trans. Image Process..

[37] Leng X., Ji K., Yang K., Zou H. (2015). A bilateral CFAR algorithm for ship detection in SAR images. IEEE Geosci. Remote Sens. Lett..

[38] Wang C., Jiang S., Zhang H., Wu F., Zhang B. (2014). Ship detection for high-resolution SAR images based on feature analysis. IEEE Geosci. Remote Sens. Lett..

